# Projecting population distribution under depopulation conditions in Japan: scenario analysis for future socio-ecological systems

**DOI:** 10.1007/s11625-020-00835-5

**Published:** 2020-08-06

**Authors:** Keiko Hori, Osamu Saito, Shizuka Hashimoto, Takanori Matsui, Rumana Akter, Kazuhiko Takeuchi

**Affiliations:** 1 United Nations University Institute for the Advanced Study of Sustainability, 5-53-70 Jingumae, Shibuya, Tokyo, 150-8925 Japan; 2 Institute for Global Environmental Strategies, 2108-11, Kamiyamaguchi, Hayama, Kanagawa 240-0115 Japan; 3 Graduate School of Agricultural and Life Sciences, The University of Tokyo, 1-1-1 Yayoi, Bunkyo-ku, Tokyo, 113-8657 Japan; 4 Graduate School of Engineering, Osaka University, 2-1, Yamadaoka, Suita, Osaka, 565-0871 Japan; 5 Institute for Future Initiatives, The University of Tokyo, 7-3-1 Hongo, Bunkyo-ku, Tokyo, 113-0033 Japan

**Keywords:** Depopulation, Population distribution, Projection model, Scenario analysis, Compact and dispersed, Socio-ecological system

## Abstract

This study develops a projection model of future population distribution on the basis of Japan’s current depopulation trend and applies this model to scenario analyses that assume population compactification and dispersion. The model enables a description of population migration at two levels. First, municipal populations are projected using the cohort-component method, and second, the spatial distribution of populations within municipalities is projected at a 500 m grid resolution with the use of the gravity model. Based on the Japanese depopulation context and the country’s National Spatial Strategy, the compact scenario predicts the formation of medium-scale regional urban areas (population centers located across Japan) and the concentration of people on high-density population areas within municipalities. Meanwhile, the dispersed scenario predicts the formation of more but smaller regional urban areas and the dispersion of the population to low-density areas. The simulated population distribution for 2050 reveals spatial change in population density and age structure, as well as an abundance of areas that were inhabited in 2015 but will be zero population areas by 2050. Overlay analysis of future land use maps and the simulated population distribution maps can contribute toward identifying areas where natural capital such as farmland and forest plantation should be managed but where there will be significant population loss by 2050.

**Electronic supplementary material:**

The online version of this article (10.1007/s11625-020-00835-5) contains supplementary material, which is available to authorized users.

## Introduction

### Background

In contrast to the population growth trend in much of the developing world, aging and depopulation have emerged as two important issues in many developed countries. According to United Nations Population Division (UNPD), “unprecedented” aging is a pervasive global phenomenon, and an increasing number of countries are experiencing population decreases because of sustained low fertility or emigration (United Nations Population Division 2019). Among the 55 countries where population shrinkage is expected, Japan faces the most serious depopulation and radical demographic transition (Eberstadt 2012; Matanle et al. 2011). It is expected that by 2065, Japan’s population will have decreased to 88 million from a population of 127 million in 2015 (National Institute of Population and Social Security Research: IPSS 2017). The causes of depopulation stem from delayed marriage and a decline in birth rate relative to the increment of working women and diversified values in life (Cabinet Office 2015). This trend has been accelerated by the outflow of young people from rural to urban areas, where women are frequently isolated and thus face difficulty in obtaining support for raising children (Japan Policy Council 2014). Depopulation has expanded from rural and remote areas to suburban and urban areas (Matanle et al. 2011), and the Ministry of Land, Infrastructure, Transport and Tourism (MLIT) estimates that 60% of inhabited areas across the country will experience 50% or greater population loss by 2050 (MLIT 2018a).

Intertwined human societies and ecosystems constitute the interdependent socio-ecological system, in which ecosystems provide necessary services for human well-being, supported by sustainable resource utilization and management by human society (Colding and Barthel 2019; Duraiappah et al. 2010). Population change is a significant indirect driver of ecosystem change, impacting the ecosystem through changes in local land use patterns (Matanle et al. 2011; MA 2005). The abandonment of land and property is inevitable in areas undergoing depopulation, leading to serious damage to natural capital (the stocks of natural resources consisting of ecosystems) that provides ecosystem services (Matanle et al. 2011; Karcagi-Kovats and Katona-Kovacs 2012). The Ministry of Agriculture, Forestry and Fisheries (MAFF 2010) and the Ministry of the Environment (MOE 2016) reported that the decrease in the number of people who can engage in the primary industries led to increased abandoned farmlands and degraded forests within the depopulated and aging rural communities of Japan. Such underuse and undermanagement of natural capitals have been recognized as crises that drive biodiversity loss and the decline in ecosystem services (MOE 2010; Ohsawa et al. 2019). Urban shrinkage has also become a significant research agenda for the social and land use sciences (Haase et al. 2012). However, if abandoned land is managed for rewilding, it is possible that such designed “new nature” can provide various ecosystem services (Gross 2008; Navarro and Pereira 2012). Thus, to plan appropriate management strategies to maintain and recreate sustainable socio-ecological systems at the local scale, projections of future population distribution with high spatial resolution are essential.

City shrinkage is a multi-layered process caused by various patterns of out-migration. Therefore, spatially estimating feasible demographic structures is problematic (Gross 2008; Feldhoff 2013). Matanle (2014) argues that internal migration in Japan, leading to both rural and urban shrinkage, can be understood at four levels of movement: inter-regional (sub-national), inter-prefectural, inter-municipal, and intra-municipal. Although complexities and uncertainties about social processes exist, an experimental approach should be used to project future population distribution and ecological design requirements (Gross 2008). Scenario analysis which seeks plausible futures with multiple assumptions about demographic and development trends has been widely recognized as an experimental approach to the development of national spatial strategies (Daly and Kitchin 2013).

Of significant importance to an exploration of the impact on ecosystems is the question of whether a shrinking society will lead to population compactification or dispersion. This question has led to dominant and contradictory theories about sustainable urban forms (Holden and Norland 2005), and can be rephrased as intensive development (or settlement) within a small area or extensive development over a large area (Soga et al. 2015). As a movement related to compactification or dispersion thus far in Japan, the continued outflow of people from rural to urban areas and expansion of urban areas, including suburban areas, have occurred due to industrialization and urbanization during the era of high economic growth after World War II (Feldhoff 2013; Matanle 2017). Relocation to outside of metropolitan areas has been promoted for industries and population to mitigate constant population loss in rural areas since the 1960s (Cabinet Office 2001). Thereafter, the necessity of development with compactification has been the subject of debate since the 2000s in response to progressive risks, such as a decline in the working population and intensification of environmental problems (Ohashi and Ishizaka 2009; Murayama 2016). The compact society and the dispersed society have different ecosystem impacts. For example, continuous forested or green spaces can be maintained under compact development, enabling the conservation of local biodiversity (Sushinsky et al. 2013; Gagné and Fahrig 2010). A dispersed society, on the other hand, provides populations with access to nature and opportunities to reconnect with nature, and promotes the utilization of local natural resources through agriculture and forestry (Scott et al. 2016; MOE 2018). Given the different impacts on socio-ecological systems, projecting population distribution under processes of compactification and dispersion is very important.

### Modeling future population distribution

A review of previous research that models future population distribution reveals a common approach to nation-wide spatial population distribution. Multiple-step simulation processes, for example, at the state or municipal level and at the grid level, have been applied to express compactification and dispersion. Wissen et al. (2010) projected future population distribution in Switzerland using four scenarios in two steps: the allocation of municipal population gain and loss, and settlement distribution modeling at 15 m resolution. The U.S. Environmental Protection Agency (EPA 2009) estimated state level populations using the cohort-component model as a first step, and then simulated spatial allocation of housing density using the gravity model at 1 ha resolution. Thorn et al. (2017) simulated decadal change in population density in New Hampshire for 2020–2100 by incorporating municipal population allocation and the allocation of 30-m grid cells using cost distance-weighted gravity model.

Although these studies share some common approaches, none had depopulation trends as a baseline assumption. Daly and Kitchin (2013) characterized decline-oriented planning as a spatially selective approach to cost-effective redevelopment, unlike the growth-oriented planning of “distribution” under circumstances of quantitative increase. Ohashi et al. (2019) argue that population decrease involves quite complex processes meaning that it cannot always be explained by the same simple mechanisms that express population growth. Given these arguments, an appropriate method must be used to project spatial population distribution trends under conditions of serious population decline in Japan.

In the Japanese context, Ariga and Matsuhashi (2012) and Matsui et al. (2019) have attempted to project population distribution by applying compact and dispersed scenarios while assuming a fundamental depopulation trend. However, these studies only modeled population distribution within municipalities or prefectures and did not adopt multi-scale simulations, such as the common processes observed in previous studies. As mentioned above, four levels of internal migration (from inter-regional to intra-municipal) have been observed to cause population decline in Japan (Matanle 2014). This suggests that developing a new projection method to express both inter-prefectural or inter-municipal and intra-municipal migration under depopulation trends would make a significant contribution to a more realistic scenario analysis of depopulation in Japan.

### Objective of this study

The aim of this study was to develop a population distribution projection model under conditions of depopulation, expressing migration at multiple levels, to conduct scenario analyses that assume compactification and dispersion of Japan’s population. This study also explored the availability of human labor for the sustainable management of natural capital, such as the maintenance of farmland and secondary forest, and this paper proposes sustainable design strategies for future socio-ecological systems in Japan. The developed model presents Japan as a “pioneer” country with crucial and ongoing depopulation challenges and aims to provide useful insights for other Asia-Pacific and European countries experiencing depopulation.

## Materials and methods

### PANCES scenarios

To explore plausible natural capital and ecosystem service futures across Japan, the PANCES (Predicting and Assessing Natural Capital and Ecosystem Services through an Integrated Social-Ecological Systems Approach) project developed four scenarios (PANCES 2017; Saito et al. 2019) using the scenario axis method (Klooster and Asselt 2006). These four scenarios were situated on two axes: (1) future society will have a “natural capital base” that promotes ecosystem-based infrastructure development and land management, or a “produced capital base” that depends more on human infrastructures and technologies; and (2) population concentration will advance toward “urban compactification”, with populations concentrated in compact cities and the rewilding of underutilized land, or there will be less concentration, leading to a decentralized society with “dispersed populations” maintaining rural communities. As part of the PANCES project, Matsui et al. (2019) developed a simulation model for population spatialization under the second axis of urban compactification versus dispersed population. This model applied a gravity-based population allocation algorithm at 1 km resolution to express migration within prefectures. To improve the reality of population distribution and spatial resolution, the study presented in this paper adopts a new process to express migration at multiple levels.

### Overview of the developed model and scenario assumptions

Under full-blown population decline, Japan’s national land concepts are as follows: “development of national land promoting interaction-led regional^1^ revitalization” and “multi-layered and resilient compact and networked structure” (MLIT 2015). These concepts present a vision of the concentration of infrastructure, services, and industries in specific areas that hold a certain population size throughout Japan. It is a vision of regional revitalization and innovation triggered by the active interaction of people, goods, money, and information from different areas, such as between urban areas and rural areas. In particular, this vision seeks to mitigate the continuous net outflow of residents from rural areas to Tokyo, where the fertility rate is lowest, as such migration has intensified rural population decline, leading to increased depopulation.

Measures to realize a multi-layered “compact and networked structure” vary depending on regional scale and characteristics (MLIT 2015). To stem population flow from rural to metropolitans, especially to the Tokyo, Osaka, and Nagoya metropolitan areas, the promotion of regional urban areas is required. Regional urban areas indicate areas outside of metropolitans becoming population or business centers around which vigorous local economic and residential bases can form. The Ministry of Internal Affairs and Communications (MIC) has already taken some steps to promote such regional centers. For example, regional cities with populations greater than 200,000 have been promoted by MIC as centers of high level service function and business called “Core Regional Urban Areas (MIC a)”, while cities with populations of more than 40,000 and surrounding municipalities have been named “Self-Support Settlement Region (MIC b)”, and proposed as centers of small clusters of settlements. It has been recommended that different scales or types of population centers are formed as regional urban areas based on existing city scales in each region. With regard to population distribution within municipalities, MLIT (2015) has proposed the formation of compact cities by concentrating urban functions and settlement within existing urban centers, and developing “small stations” in rural areas that will provide life-services to surrounding communities. In addition, a movement to promote a return to rural living, including in depopulated areas, has also been suggested as an alternative approach to stem depopulation.

**Fig. 1:**
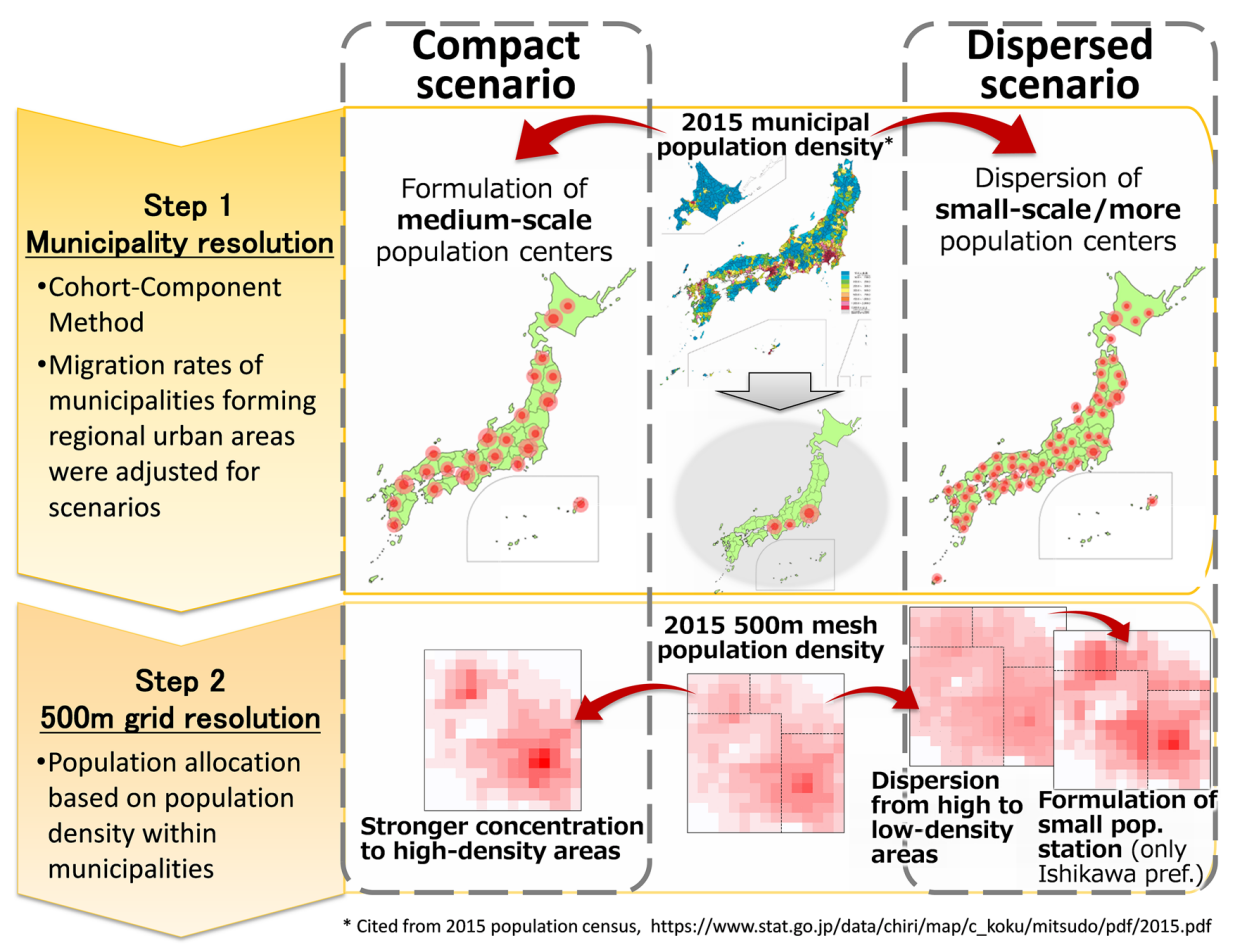
Overview of model structure and images of scenario assumptions

Given these national strategies, this study developed a projection model of Japanese population distribution using two projection steps (Fig. 1).

The first municipality resolution model expressed different formations of population centers as realistic scenarios under depopulation conditions. New migration trends were assumed to occur beyond prefectural boundaries and sub-national blocks. In the compact scenario, this study assumed medium-scale (smaller than major metropolitan) regional urban areas with populations of more than 200,000, corresponding to “Core Regional Urban Areas” (MIC a). In the dispersed scenario, municipalities of more than 40,000 were assumed to form smaller but more regional urban areas, corresponding to “Self-Support Settlement Region” (MIC b). Based on the U.S. Environmental Protection Agency (EPA 2009) model, an adjusted cohort-component model was applied to the municipality resolution model.

The second step of the model reproduced the population distribution at a 500 m grid resolution by population density projections using the arranged gravity model based on previous research (EPA 2009; Thorn et al. 2017; Matsui et al. 2019). In this study, the compact scenario assumed population concentration in high-density areas within municipalities. In the dispersed scenario, the dispersion or return of people to low-density areas was assumed in the nation-wide simulation. By way of prefectural case study, a further analysis of the formation of small population centers in rural areas to maintain community functions was conducted.

In the final step, an overlay analysis of projected population distribution and future land use and land cover (LULC) map was conducted to spatially examine the availability of labor for the management of natural capital, such as the maintenance of farmland and secondary forests. This analysis was conducted so that the projected population maps can contribute to the design of appropriate sustainable socio-ecological strategies.

### Municipality resolution population distribution model and scenario assumption

As with the PANCES scenarios (Saito et al. 2019), population distribution projections were conducted to 2050, based on data from Japan’s 2015 national population census. The municipal population was projected according to sex and 5-year age groups, using the cohort-component method with 5-year steps as shown in Eqs. (1) and (2), referring to Regional Population Projections for Japan: 2015–2045 (IPSS 2018):

1 $$P_{t, i,j,k } = \left( {s_{t - 5 \to t, i - 1 \to i, j,k} + m_{t - 5 \to t, i - 1 \to i, j,k} } \right)*P_{t - 5,i, j,k } \ldots \left( {i = 2\sim 18} \right),$$

2 $$P_{t, i,j,k } = c_{t,k} *b_{t, j,k} *\mathop \sum \limits_{i = 4}^{10} P_{t,i, j = 2,k } \ldots \left( {i = 1} \right),$$

where *P*_*t,i,j,k*_ is the population of municipality *k* (1–1799, for each city, town, village, special ward, and administrative district of ordinance-designated cities^2^) in year *t* (2020–2050), by 5-year age group *i* (*i *= 1 [age 0–4]–18 [over age 85]) and sex type *j* (*j *= 1 [male] and 2 [female]). *s*_*t*−5*→t,i*−1*→i,j,k*_ (0 ≤ *s* ≤ 1) and *m*_*t*−5*→t,i*−1*→i,j,k*_ (− 1 ≤ *m*) express survivorship rates in municipality *k* and net migration rates to municipality *k* of 5-year age group *i* − 1 from year *t* − 5 to year *t* by each sex type *j*. A minus *m* value means the outflow of population from municipality *k* exceeds inflow. *c*_*t,k*_ and *b*_*t,j,k*_ are, respectively, child–woman ratios (average population of the age group 0–4 per woman in the age group 15–49) and sex ratios for the population of age group 1 [age 0–4] of municipality *k* in year *t*. The 2015 baseline municipal population and the parameters to project future population (*s*, *m*, *c*, *b*) were cited from the 2015 Population Census (Statistics Bureau of Japan: SBJ 2016) and Regional Population Projections for Japan: 2015–2045 (IPSS 2018). The 2040 to 2045 parameters (IPSS 2018) were utilized again to project the 2045 to 2050 municipal population.

Municipal migration rates were adjusted for each scenario. In the compact scenario, by referring to the requirements and actual examples of a central city of “Core Regional Urban Areas” (MIC a), municipalities with populations greater than 200,000 and daytime/night-time population ratios (SBJ 2016) of more than 0.98 were selected as regional urban centers. Ordinance-designated cities were judged by the total population of administrative districts constituting the city. In contrast, based on the central city’s requirements of “Self-Support Settlement Region” (MIC b), municipalities located outside of Japan’s three major metropolitan areas, with populations of more than 40,000, and with daytime/night-time population ratios of more than 1 were selected as regional urban centers in the dispersed scenario.

These central cities and their adjacent municipalities (described as municipality group A) were assumed to form regional urban areas, and the migration ratios of these municipalities were enhanced according to the following equation:

3 $$\dot{m}_{t - 5 \to t, i - 1 \to i, j,k \in A} = m_{t - 5 \to t, i - 1 \to i, j,k \in A} + \left( {\alpha - 1} \right)*\left| {m_{t - 5 \to t, i - 1 \to i, j,k \in A} } \right|,$$

where $\dot{m}$_*t*−5*→t,i*−1*→i,j,k∈A*_ is adjusted migration rate of 5-year age group *i* − 1 to municipality *k∈A* from year *t* − 5 to year *t* by each sex type *j*. *α* (*α *> 1) is a parameter for each scenario to determine the strength of population concentration in regional urban areas.

Increased population flow into group A municipalities was assumed to come from municipalities not included in group A. The migration rates of these other municipalities were decreased according to the following equations:

4 $$\beta_{t - 5 \to t, i - 1 \to i, j} = \frac{{\left( {\mathop \sum \nolimits_{k \in A} \left( {\alpha - 1} \right)*\left| {m_{t - 5 \to t, i - 1 \to i, j,k} } \right|*P_{t - 5,i, j,k } + \mathop \sum \nolimits_{k \notin A} \left| {m_{t - 5 \to t, i - 1 \to i, j,k} } \right|*P_{t - 5,i, j,k } } \right)}}{{\mathop \sum \nolimits_{k \notin A} \left| {m_{t - 5 \to t, i - 1 \to i, j,k} } \right|*P_{t - 5,i, j,k } }},$$

5 $$\dot{m}_{t - 5 \to t, i - 1 \to i, j,k \notin A} = m_{t - 5 \to t, i - 1 \to i, j,k \notin A} + \left( {1 - \beta_{t - 5 \to t, i - 1 \to i, j} } \right)*\left| {m_{t - 5 \to t, i - 1 \to i, j,k \notin A} } \right|,$$

where $\dot{m}$_*t*−5*→t,i*−*1→i,j,k∉A*_ is adjusted migration rate of 5-year age group *i* − 1 to municipality *k∉A* from year *t* − 5 to year *t* by each sex type *j*. *β*_*t*−*5→t,i*−*1→i,j*_ (*β *> 1) is a dependent variable calculated by *α* to express the degree of additional population outflow from the municipalities outside of the regional urban areas.

Considering the significance of expressing diverse of scenario assumptions, including extreme and plausible population distribution (Matsui et al. 2019), extreme and middle degree population concentrations into regional urban areas were expressed by adjusting parameter *α*. Table 1 summarizes the municipality resolution scenario assumptions.

**Table 1:**
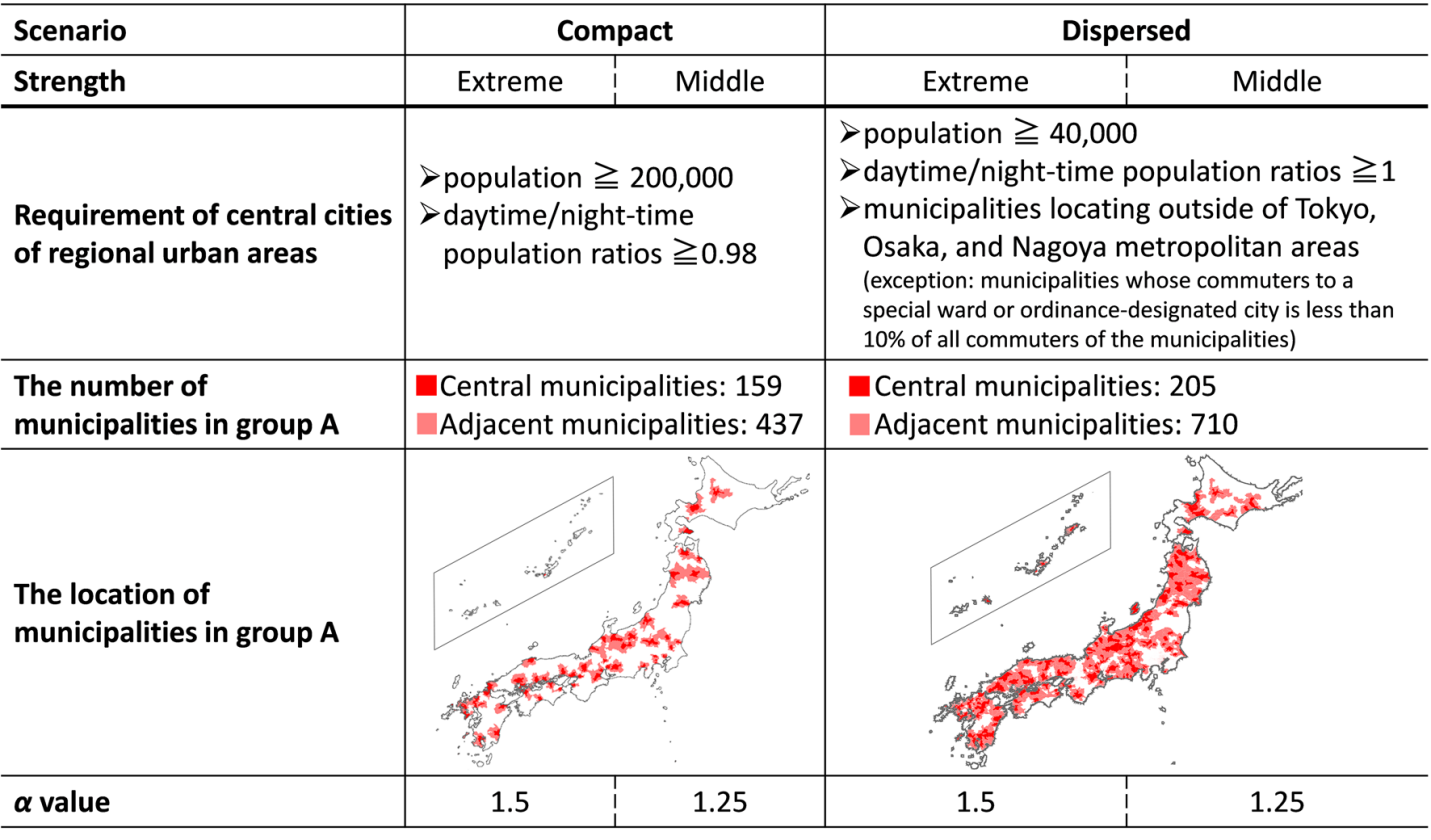
Summarized scenario setting according to the municipality resolution

### Grid resolution population distribution model and scenario assumption

#### Mathematical model for nation-wide projection

The grid resolution nation-wide population distribution was projected by allocating projected municipal populations into grids based on population density, utilizing, as base data, the 2015 population density in 500 m grid resolution established by SBJ (2017). The number of populated grids in 2015 was 471,066. Each of these grids was targeted as an inhabited grid, the 2050 population under the two scenarios was calculated according to the following equation:

6 $$p_{2050, i,j,k,l } = P_{2050,i, j,k } * r_{2050, i,j,k,l} ,$$

where *p*_2050,*i,j,k,l*_ and *r*_2050*,i,j,k,l*_ express the population of grid *l* belonging to municipality *k* and the population ratio of grid *l* to total population of municipality *k* in 2050, by 5-year age group *i* and sex type *j*.

*r*_2050*,i,j,k,l*_ was calculated corresponding to each scenario assumption. In the compact scenario, concentration in densely populated areas within municipalities was assumed, and in the dispersed scenario, dispersion to low-density areas within municipalities was assumed. For each municipality, age group, and sex, grids with an expected increased population ratio (grid group [*a*]) were extracted. Grids with a higher ranked population ratio in 2015 were selected as grid group [*a*] for the compact scenario, and those with lower ranked population ratio (grids with accumulated population ratios up to *γ*(0 < *γ* < 1) were selected as grid group [*a*] for the dispersed scenario. *γ* is a threshold for each scenario to determine which grids are expected to experience an additional inflow of population; outflow of population is assumed in the others. *r*_2050*i,j,k,l∈a*_ was calculated according to the following equation:

7 $$r_{2050, i,j,k,l \in a} = r_{2015, i,j,k,l \in a} *\delta ,$$

where *δ* (1 < *δ*) is a parameter for each scenario to determine the increment degree of population ratio of grids included in group [*a*]. The population ratios of grids not included in group [*a*] were evenly decreased according to the following equations such that the population ratio could be totaled to 1 within the municipality:

8 $$\varepsilon_{ i, j,k} = \frac{{1 - \mathop \sum \nolimits_{l \in a} r_{2050, i,j,k,l \in a} }}{{1 - \mathop \sum \nolimits_{l \in a} r_{2015, i,j,k,l \in a} }},$$

9 $$r_{2050, i,j,k,l \notin a} = r_{2015, i,j,k,l \notin a} *\varepsilon_{ i, j,k} ,$$

where *ε*_*i,j,k*_ (0 ≤ *ε* ≤ *1*) is a dependent variable expressing the decrement degree of population ratios of grids not included in group [*a*] for each municipality *k*, age group *i*, and sex type *j*.

The parameters for all scenarios in this grid resolution model are summarized in Table 2. With regard to *γ*, a larger value was set in the compact scenario than in the dispersed scenario. This is because the population is unevenly distributed and concentration to a limited area (Ariga and Matsuhashi 2012) and, in general, the cumulative probability of population density shows a bow-shaped curve extending to the lower right [the Lorenz curve (Lorenz 1905)]. The compact scenario had a larger *γ* threshold because the number of grids in which population ratio increased was less than in the dispersed scenario when the same thresholds were used. Consistency between scenarios was maintained by the total number of population ratio with changed distribution [calculated by multiplying *γ* and (*δ* − 1)].

**Table 2:** Summarized scenario setting for nation-wide projection according to the grid resolution

Scenario	Compact		Dispersed	
Strength	Extreme	Middle	Extreme	Middle
Requirement of grid group [a]	Higher ranked grids on 2015 population ratio in municipality (until accumulated population ratio reaches *γ*)		Lower ranked grids on 2015 population ratio in municipality (until accumulated population ratio reaches *γ*)	
*γ* value	0.4		0.2	
*δ* value	1.5	1.25	2	1.5

Additional processing was conducted at the end of the grid resolution projection to enable comparison with the business as usual (BAU) scenario for 2050 projected by MLIT (2018b). The population of grids projected to be inhabited by very small populations was replaced with 0 using the following equation, based on MLIT (2018b):

10 $$\dot{p}_{{2050, i,j,k,l,l^{{\prime }} }} = \left[ {\begin{array}{*{20}c} {0 \left( {{\text{if}} \mathop \sum \limits_{{l,l{\prime } = \ddot{l}{\prime }}} p_{{2050, i,j,k,l,l^{{\prime }} }} < 1} \right)} \\ {p_{{2050, i,j,k,l,l^{{\prime }} }} \left( {{\text{if}} \mathop \sum \limits_{{l,l{\prime } = \ddot{l}{\prime }}} p_{{2050, i,j,k,l,l^{{\prime }} }} \ge 1} \right)} \\ \end{array} ,} \right.$$

where $\dot{p}$_2050*i,j,k,l,l′*_ is the population after the replacement process in the 500-m grid *l* constituting 1-km grid *l′* (each 1-km grid consists of four 500-m grids) belonging to municipality *k* in 2050, by 5-year age group *i* and sex type *j*. Those 500 m grids where the population of all age groups and both sex types were replaced by 0 were considered “zero population grids”. After processing, the population of grids not judged to be “zero population grids” were adjusted to fit the total municipal population for 2050.

#### Mathematical model for an additional projection, focusing on Ishikawa prefecture

In addition to the nation-wide projection, an additional projection for the dispersed scenario was conducted that focused on Ishikawa prefecture. Although flat dispersion of the population to low-density areas was expressed in the nation-wide grid resolution model, the Japanese government has proposed the formation of “small stations” as central communities to maintain the vital life services in rural areas. Under this strategy, population concentration in these small stations is expected to be a probable and realistic trend. According to the Cabinet Office (2017), small stations should be formulated in each elementary school district or each former municipal area. In the 1880s, 1950s, and 1990s–2000s, Japan experienced dramatic mergers of its municipalities (MIC c). The significance of a multi-locational system that maintains functions in former municipal centers has been suggested as a means to prevent drastic population outflow from rural areas (Buhnik 2017). Considering this context, population distribution with the formation of a population center in each former municipal area was projected under the dispersed scenario. This additional model is hereafter referred to as the former municipality model.

**Fig. 2:**
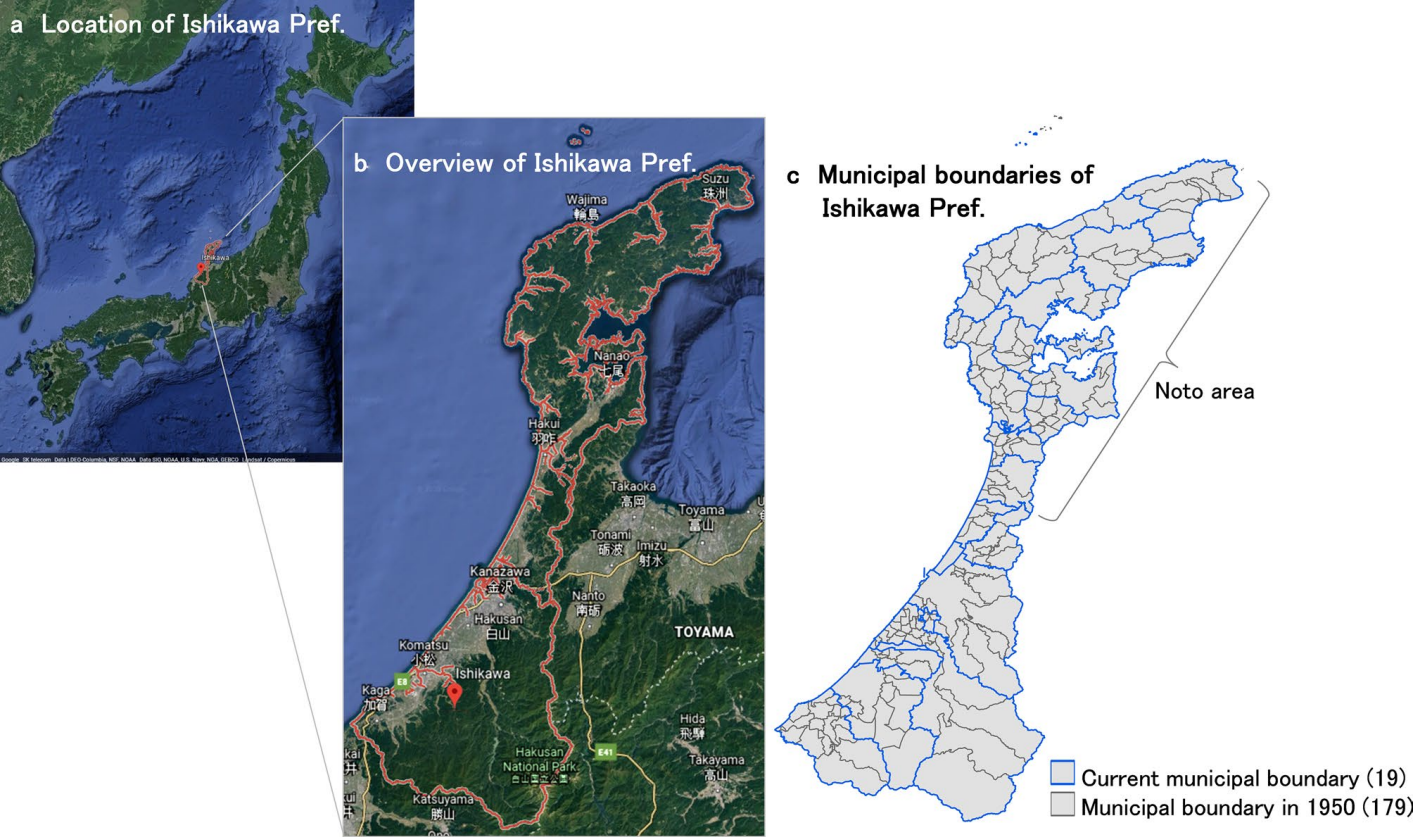
Location, overview, and municipal boundaries of Ishikawa prefecture

Ishikawa prefecture was targeted for the application of the former municipality model. This prefecture is located on the Sea of Japan coast, in the middle of Honshu, Japan’s main island (Fig. 2). The prefecture’s 19 municipalities vary in type (Ishikawa Prefecture a), and include Kanazawa city, with a population of more than 460,000 (Kanazawa city), and the rural municipalities on the Noto Peninsula, which have been selected by the Food and Agriculture Organization of the United Nations (FAO) as a Globally Important Agricultural Heritage System (FAO). Ishikawa prefecture is a case study site of the PANCES project. Although Hashimoto et al. (2019) have attempted a scenario analysis of land use and ecosystem services on Noto Peninsula, population distribution did not fully consider the local demographic situation. Given this background, Ishikawa prefecture was selected as a case study site to explore the former municipality model.

Using 2015 data, 5581 populated grids in Ishikawa prefecture were targeted as grids into which future populations could be allocated. The boundary data of former municipalities were obtained from National Land Numerical Information, Administrative Zones Data (MLIT 2019). The 179 municipal boundaries in place in 1950 were used as the boundaries of former municipalities (see Fig. 2). This additional projection was conducted only for the dispersed scenario based on the nation-wide projection of the 2050 population at the 500 m grid resolution (*p*_2050_ in Eq. (11)). This model expressed the concentration of flatly dispersed population in the central areas of former municipalities with no change to the total population of former municipal areas, according to the following equations:

11 $$p{\prime }_{{2050, i,j,k^{{\prime }} ,l }} = \mathop \sum \limits_{{l,k^{{\prime }} = \ddot{k}^{{\prime }} }} p_{{2050, i,j,k^{{\prime }} ,l }} * r{\prime }_{{2050, i,j,k^{{\prime }} ,l}} \left( {k^{\prime} = 1, 2, \ldots \ddot{k}^{{\prime }} \ldots 179} \right),$$

12 $$r^{{\prime }}_{{2050, i,j,k^{{\prime }} ,l \in a^{{\prime }} }} = r^{{\prime }}_{{2015, i,j,k^{{\prime }} ,l \in a^{{\prime }} }} *\delta^{{\prime }} \text{,}$$

13 $$\varepsilon^{{\prime }}_{{ i, j,k^{{\prime }} }} = \frac{{1 - \mathop \sum \nolimits_{{l \in a{\prime }}} r^{{\prime }}_{{2050, i,j,k^{{\prime }} ,l \in a^{{\prime }} }} }}{{1 - \mathop \sum \nolimits_{{l \in a{\prime }}} r^{{\prime }}_{{2015, i,j,k^{{\prime }} ,l \in a}} }},$$

14 $$r^{{\prime }}_{{2050, i,j,k^{{\prime }} ,l \notin a^{{\prime }} }} = r^{{\prime }}_{{2015, i,j,k^{{\prime }} ,l \notin a^{{\prime }} }} *\varepsilon^{{\prime }}_{{ i, j,k^{{\prime }} }} ,$$

where *p′*_2050*,i,j,k′,l*_ and *r′*_2050*,i,j,k′,l*_ express the population of grid *l* belonging to former municipality *k′* and the population ratio residing in grid *l* to the total population of former municipality *k′* in 2050, by 5-year age group *i* and sex type *j*.

As grids with concentrated population were named grid group [*a′*] in the former municipality model, higher ranked grids in population ratio *r′* [grids with a accumulated population ratio of no more than *γ′*(0 < *γ′* < 1)] in 2015 were selected for each former municipality. *γ′* is the threshold dividing the grids in group [*a′*] from others within the former municipality, and *δ′*(1 < *δ*) expresses the incremental degree of population ratio of grids included in group [*a′*]. As *γ′* and *δ′*, 0.4 and 1.5 were set for both extreme and middle cases. Finally, the same replacement process as shown in Eq. (10) was conducted.

### Overlay of the projected population distribution map and the future land use map

As argued in “Background” , depopulation in areas holding natural capitals, such as agricultural land and forests, causes a decrease in labor to maintain and manage such capitals, which leads to their underuse or abandonment (MAFF 2010; MOE 2016). The locations where such problems are likely to occur are dependent on future population distribution and land use. Thus, the study spatially examined the labor availability for natural capital management by overlaying the projected population distribution map and future land use map.

2050 LULC maps, developed according to Shoyama et al.’s (2019) method, were used to conduct the overlay analysis. The maps correspond to four PANCES scenarios at the 500 m grid resolution. The data consisted of 10 categories: residential area, paddy field, cropland, other agriculture lands, abandoned farmland, grassland and bush, natural forest, secondary forest, plantation, and others. The 2050 LULC maps of the “Natural capital-based compact society (NC)” scenario and the “Produced capital-based compact society (PC)” scenario were overlaid by the 2050 population map of the extreme case compact scenario, while the LULC maps of “Natural capital-based dispersed society (ND)” scenario and “Produced capital-based dispersed society (PD)” scenario were overlaid by the map of the extreme case dispersed scenario.

The land use category occupying the largest area of each 500-m grid of the population map was identified for each of those grids as their land cover attribute. Among the categories on the LULC map, paddy field, cropland, other agriculture lands, grassland and bush, secondary forest, and plantation were considered to be lands in need of human management [hereafter referred to as MNC (managed natural capital lands)]. Across the country, scenario differences for populations living in the grids covered by the MNC were analyzed to provide an overview of changes in labor availability for the management of local natural capital. MNC grids projected to have high levels of depopulation (particularly zero population grids) and aging (more than half of residents aged over 65) were spatially identified for Ishikawa prefecture.

## Results

### Population distribution

#### Nation-wide projection

**Fig. 3:**
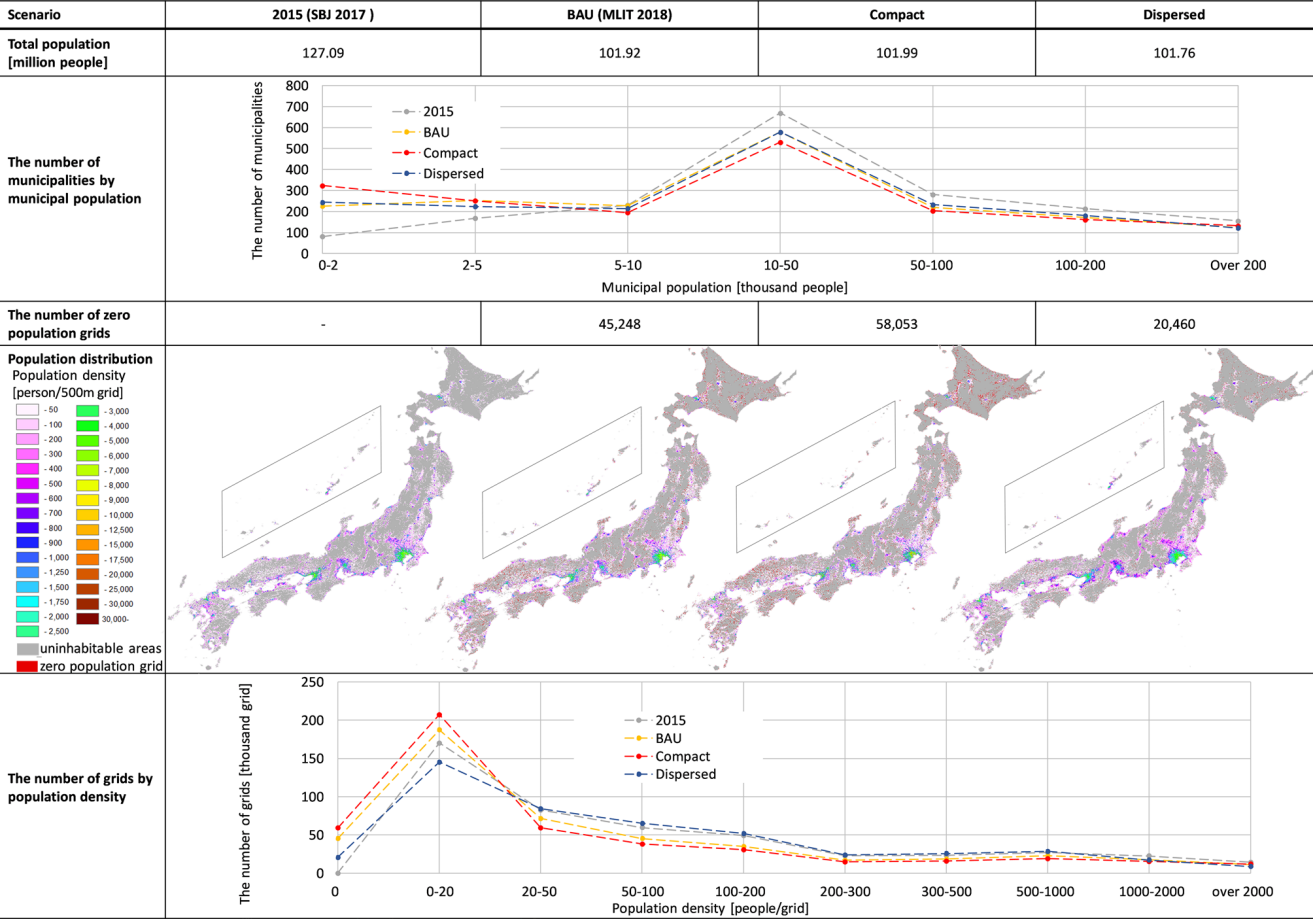
Summarized results of nation-wide population distribution projection

The resulting population distribution and statistical data for each scenario are summarized in Fig. 3. Due to limitations of space, only extreme cases are described, with medium-range cases provided as supplemental materials in Online Appendix 1.

Total population resulted in an increment of approximately 70,000 in the compact scenario, and a decrement of 160,000 in the dispersed scenario. This was derived from differences in original migration rates by type of municipality. The population of each municipality was calculated by Eq. (1). The population of larger municipalities with comparatively higher migration rates increased in the compact scenario, and the population of smaller municipalities with lower migration rates, including minus values, increased in the dispersed scenario. That is the reason why total population under scenarios differed from BAU. These differences can be interpreted as the rise and fall of population flow between Japan and other countries.

As the third row of Fig. 3 shows, the compact scenario resulted in more polarized population sizes in municipalities, with an increase in the number of municipalities of less than 2000 and those of more than 200,000, while the number of mid-sized municipalities decreased compared to BAU. In the dispersed scenario, a slight increase in the number of municipalities with more than 50,000 and less than 200,000 was observed.

In the grid resolution, the number of zero population grids increased by approximately 28% from BAU in the compact scenario and decreased by 54% in the dispersed scenario. Zero population grids tended to appear on the fringes of inhabited area, and were especially prevalent in the Hokkaido block and the Chugoku block in the compact scenario (fifth row of Fig. 3). The formation of smaller population centers across Japan were reproduced in the dispersed scenario. The number of grids with a population density of less than 20 and more than 2000 increased in the compact scenario compared to the BAU (sixth row of Fig. 3). In contrast, under the dispersed scenario, population density could be maintained at the medium level, i.e., with more grids in the 20–2000 people/grid range compared to BAU.

#### Ishikawa prefecture

**Fig. 4:**
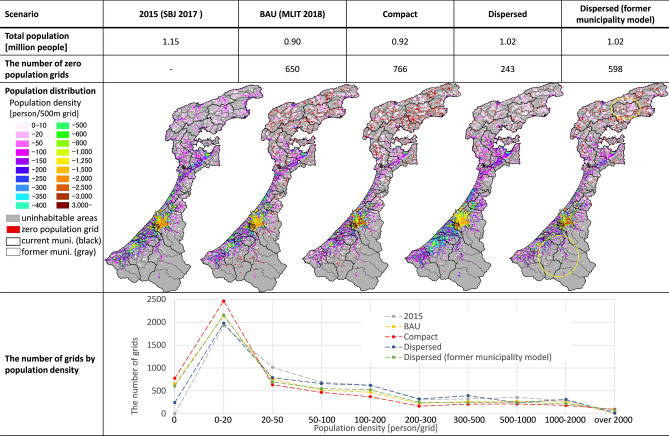
Summarized results of population distribution in Ishikawa prefecture

The results of statistical analysis of Ishikawa prefecture’s population distribution, including the outcome of the former municipality model, are summarized in Fig. 4. The number of zero population grids increased by approximately 18% from BAU in the compact scenario and decreased by approximately 63% in the dispersed scenario. In the former municipality model dispersed scenario, the number of zero population grids was also less than for BAU or the compact scenario.

Only Kanazawa city and its adjacent municipalities were selected as the regional urban area to form a population center in the compact scenario, while in the dispersed scenario, Nanao city and its adjacent municipalities on Noto Peninsula (location is shown in Fig. 2b) were also assumed to form regional urban areas. Nanao city and its surrounding areas maintained a larger population in the dispersed scenarios compared to the compact scenarios (fourth row of Fig. 4). In areas indicated by yellow circles, the former municipality model resulted in comparatively higher density grids in each former municipality area. For Kanazawa city, urban residential sprawl is a serious issue for city planners (Ishikawa Prefecture 2018). Thus, the results of progressive concentration in the city center under the compact scenario and former municipality model dispersed scenario were deemed more desirable.

The number of grids by population density resulting from the former municipality model was positioned between the compact and dispersed scenarios for nation-wide projection and was close to the BAU result (bottom of Fig. 4). However, the number of grids with a density between 50 and 300 people/grid was larger than that of the BAU, reflecting the maintenance of smaller population centers.

### Overlaying on the future land use map

**Fig. 5:**
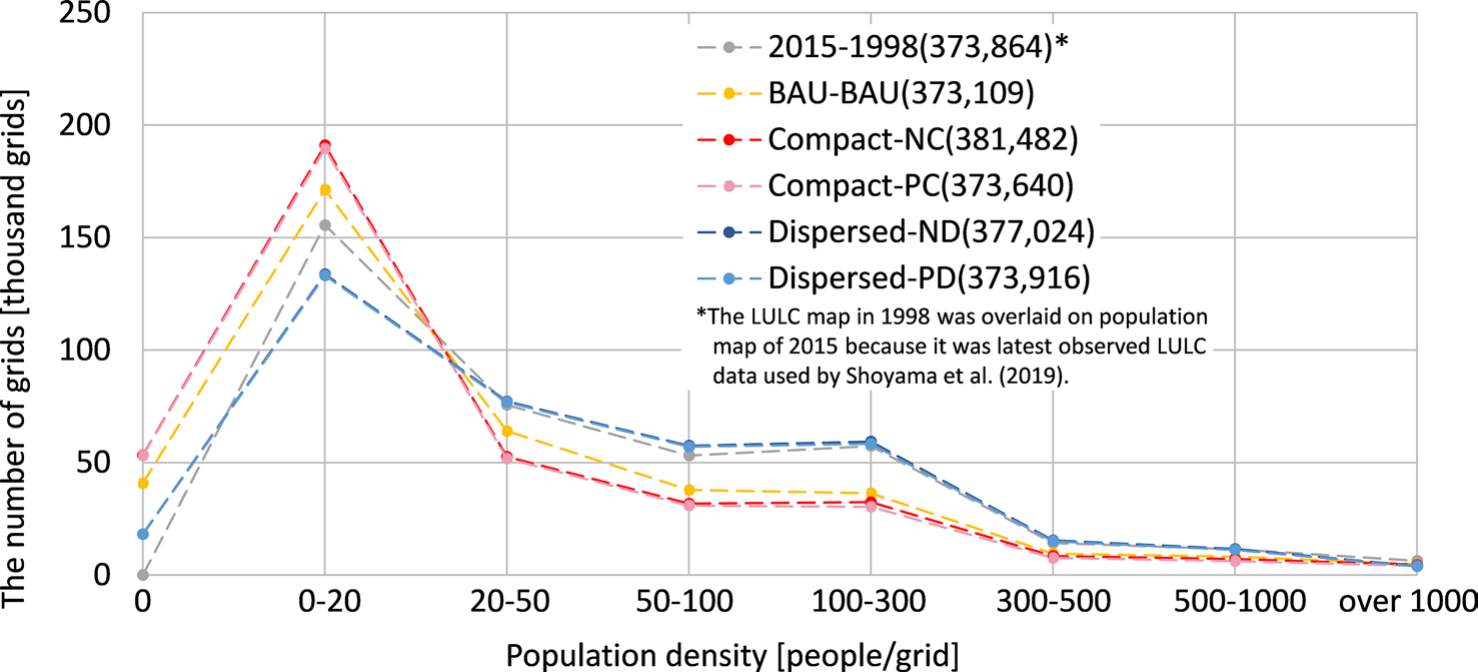
The number of grids covered by MNC by population density (nation-wide)

Based on the nation-wide results of the overlay analysis, Fig. 5 shows the number of grids covered by MNC by population density. The legend indicates the two scenarios, namely overlaid population distribution map and LULC map. The reason for the change in the number of grids is due to changes to certain types of land use, such as paddy fields or plantation forests, based on scenario assumptions [see Shoyama et al. (2019) for details]. The grids which were intended to hold a population of only 0–20 people and be covered by the MNC were concerned to face possible shortages in labor required to maintain MNC. In the compact scenario, the number of such grids was larger than those of other scenarios and resulted in nearly 250,000 grids. In the dispersed scenario, there were a greater number of grids covered by MNC with populations of 20–300 people, compared to either the compact scenario or BAU. As a nation-wide trend, the risk of shortage in labor for the management of natural capitals was deemed higher in the compact scenario compared with the dispersed scenario.

**Fig. 6:**
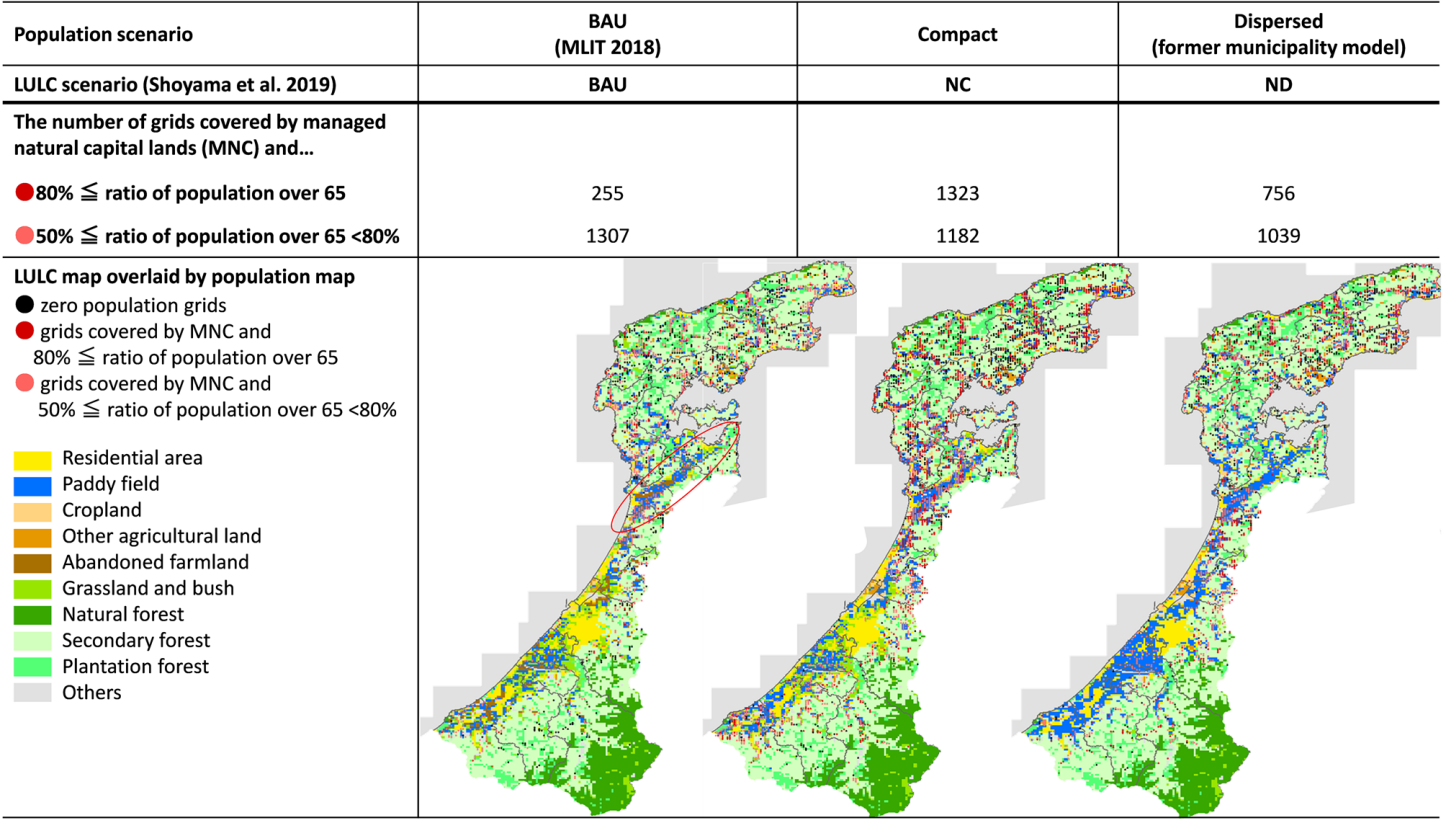
Summarized results of overlaying analysis focusing on Ishikawa prefecture

An example of the spatial results of overlay analysis is shown here, with a focus on Ishikawa prefecture. The overlaid results for natural capital-based scenarios are provided in this section (Fig. 6), and the results of produced capital-based scenarios are provided in Online Appendix 2. The maps in Fig. 6 show the spatial distribution of 10 land cover types, and the depopulated grids (black dots) and highly aging grids (red and pink dots) covered by MNC. The number of grids with red dots increased in both the compact and dispersed scenarios, but the increment was more dramatic in the compact scenario (third row of Fig. 6). In the compact scenario, almost all the grids covered by MNC in the Noto area (the location is shown in Fig. 2) were expected to experience serious depopulation and aging.

In addition, the different scenarios revealed differences in type of MNC that would be expected to face higher levels of depopulation and aging. For both scenarios, plantation forest was a major MNC dotted by red and pink. However, in the compact scenario, many of the grids covered by paddy field in 2050 also face aging, such as around the reclaimed land of Ouchigata (shown as a red circle in Fig. 6, Noto Digital Archive Corporation). Natural capital-based scenarios for the LULC map assumed that naturally produced rice would be preferred and larger areas of paddy field would not be abandoned compared to BAU (Shoyama et al. 2019). Ouchigata reclaimed land was also projected to be maintained as paddy fields, but in the compact scenario, more than half of the residents of this area were projected to be over 65 in 2050. This was due to the expected outflow of younger people to the mid-scale regional urban areas, such as to Kanazawa city from Hakui city or Nanao city which Ouchigata reclaimed land belongs.

## Discussion

### Methodological significance

The aim of this research was to develop a projection model of population distribution that expressed multi-level migration and assumed a depopulation trend. The model was built integrating cohort-component method with municipal resolution and a gravity model in grid resolution. This research is methodologically significant in three ways.

First, this projection model is capable of expressing all four levels of migration (inter-regional, inter-prefectural, inter-municipal, and intra-municipal) that cause depopulation (Matanle 2014). This is achieved by combining municipal resolution projections to reproduce migration beyond prefectural and sub-national block boundaries, and the grid resolution model. Given previous research in Japan (see “Modeling future population distribution”), this is a significant advance, as the model enables a realistic and complex projection of population distribution for countries experiencing depopulation. Moreover, the model expresses population increments and decrements for medium-sized municipalities and grids with medium population densities for each scenario (Fig. 3). Due to the decrease in pressure to develop land that accompanies population decline, “selective national land utilization” is advocated in Japan (MLIT 2015). This involves combining different development strategies according to municipal or area characteristics, such as compact city strategies in more populated and accessible areas, and rewilding strategies (Gross 2008) in highly depopulated and aging areas. To select the most appropriate strategy, future dynamic changes of municipalities and grids with medium-scale population density must be projected and considered. This model can provide outcomes which contribute to “selective” national land development design and local development strategies.

Second, by applying the cohort-component method, the model enables population projection by 5-year age group. This overcomes one of the limitations of Matsui et al. (2019) in which the total population was projected based on PANCES scenarios. Aging has as serious an impact on the socio-ecological system as depopulation, and spatial analysis of the degree of aging (Fig. 6) by age-specific projection is a novel and necessary approach to understanding current patterns of depopulation and aging. In addition, by adjusting parameters used for the cohort-component method, other alternative scenario analyses can be more easily conducted. Due to highly uncertain future population development (Gross 2008), a greater variety of scenarios must be examined, including the multiple fertility and mortality sets assumed by IPSS (IPSS 2017). This flexibility allows this projection model to expand scenario analysis and to apply analysis to other countries experiencing similar post-growth development pathways and eventual depopulation (Eberstadt 2012; Matanle 2017).

Third, overlaying this analysis on future LULC maps is a valuable method for determining coherent socio-ecological systems policies (see “Overlaying on the future land use map”). Decline-oriented planning is characterized by a spatially selective approach, and a coherent and holistic approach that addresses all economic, social, and environmental issues is essential (Daly and Kitchin 2013). In this research, overlay analysis spatially demonstrated how the consistency of land use as an environmental aspect and population as a social aspect can be realized. Further scenario assessments of economic aspects are expected in the future research through the integration of related models, such as SURQUAS (Smart Urban area Relocation model for sustainable QUAlity Stock, Togawa et al. 2009) or ExSS model (a regional input–output model, Gomi et al. 2011).

As other future research agenda, additional projection of future land use harmonized with this study’s projected population distribution is also significant. By utilizing the population-projection-assimilated predictive land use modeling (PPAP-LM) approach applied by Ohashi et al. (2019), actual land use patterns affected by population dynamics can be projected in order to support more realistic policy-making. Furthermore, in reference to previous research (e.g., Matsui et al. 2019), expanding the target of this projection model to the working population employed in industrial sectors related to natural capital management is also expected to support the development of feasible industrial policies.

### Policy implications

The study highlighted challenges and proposed necessary interventions to realize a sustainable socio-ecological system under the compact and dispersed population dispersion scenarios. The first potential problem of the compact scenario is that many municipalities are concerned about the loss of necessary daily services due to population decline. For example, the population size of a municipality should exceed 2500 to enable the placement probability of book stores or dental clinics to exceed 80% (MLIT 2014). Given the large number of municipalities with populations of less than 2000 among scenarios (Fig. 3), enhancing the “networks” between municipalities at various levels is more significant in the compact scenario. In this manner, healthy and cultural lives in small municipalities can be maintained. In other words, inter-municipal cooperation, which is a characteristic of decline-oriented planning (Daly and Kitchin 2013), is more significant in a compact society. “Networks for a new era” (MLIT 2015), such as advanced information and communications networks that enable remote medical care and education, must be promoted as part of the Information and Communications Policy of Japan (MIC 2019).

The second expected challenge under the compact scenario is the shortage of labor for the management of natural capitals. The overlay analysis of the grid resolution identified places where MNC should be maintained, such as paddy fields. Under the NC scenario, the identified areas, such as the Ouchigata reclaimed land, will face severe aging by 2050 (Fig. 6). To sustain the paddy fields, which are significant wintering sites for migratory birds (Noto Digital Archive Corporation), external participants in activities designed to manage MNCs are necessary. Relevant policies have been formulated to promote such a “related population” (i.e., people who continuously have relationships with a specific region; Naito et al. 2019). The initiative to call for volunteer workers for agricultural activities from outside Ishikawa prefecture is called “work stay at Ishikawa” (Ishikawa prefecture b). The national policy called “Related Population Creation and Expansion Project (MIC d)” aims to provide financial support for the local development of the creation of a related population. Moreover, smart agricultural technologies (MAFF 2019) can be considered another essential solution to support limited labor. Technologies that make activities to maintain MNCs easier and more accessible for anyone, such as power-assisted suits, must be implemented under the NC scenario. “Project for Accelerating Installation of Smart Agriculture (MAFF)” is expected to expand as a supportive policy for local efforts to install smart agricultural technologies.

As opposed to not only the compact scenario but also BAU, the dispersed scenario resulted in a larger population size in areas covered by the MNC (Fig. 5; less grids with less than 20 people) compared to 2015. In other words, the dispersed scenario cannot be achieved unless a new type of migration opposite of the current trend toward BAU becomes a major trend. This challenge remains for the dispersed scenario. The argued major keys to increase the influx of younger migrants from metropolitan areas to small regional urban and rural areas are profitable and attractive employment and attractive living environment, where raising children is easy (Cabinet Office 2019). Several measures are expected to create employment. For example, promoting the development of industries with a high affinity for localization, such as welfare, health care, interpersonal services, environmental sectors, culture, and agriculture is important. Another measure is that financial support can be given for migrants who contribute to activities for local development, such as the “Local Vitalization Cooperator,” which has been in operation since 2009 (MIC e, Hiroi 2019). Moreover, advanced measures that overcome the challenges of existing childcare and education support are necessary. As an alternative, a variety of childcare services can be promoted for migrants without relatives in the neighborhood (Kukimoto 2016). Moreover, development of rural communities with social inclusiveness throughout cultural opportunities can be expected as an effective measure (Hirata 2018).

The COVID-19 pandemic in 2020 has led to increased awareness of the risks of living in urban areas with large populations, whereas the advantages of a regional dispersed society have been discussed more seriously and widely (MOE 2020; National Governors’ Association 2020). The rapid spread of teleworking and increased number of metropolitan residents interested in migrating to rural areas have also been reported as part of the impacts of COVID-19 (Cabinet Office 2020; Nippon Institute for Research Advancement 2020). This movement can become an opportunity to accelerate the formation of a dispersed scenario. Further projection of population distribution reflecting new dispersed settlement styles and industrial structures corresponding to coexistence with COVID-19 is required in future studies.

Moreover, this study contributes to providing detailed possibilities for the mixing of multiple scenarios. As Murayama (2016) argues, there is no universal approach to land use planning for a depopulating and aging society and, in reality, a one-size-fits-all scenario cannot be rolled out across Japan. Local municipalities and communities need to plan the most appropriate direction for future growth given their unique local characteristics and circumstances and referring to the four quadrants of the PANCES scenario. The result of the study can support such initiatives through the spatial description of the different potential challenges related to population distribution under different scenarios. In addition, given that many solutions for sustainability are a combination of both nature-based and human/industrial elements (Schaubroeck 2018), future strategies in any one area must also combine natural capital and produced capital-based scenarios. One example of this is that smart agriculture technologies are recommended for Ouchigata reclaimed land to optimize a natural capital-based compact society. This exploratory analysis can lead the way to actual future strategy design by mixing PANCES scenario assumptions.

## Conclusion

This study developed a projection model of future population distribution under current Japanese depopulation trends and applied it to a series of scenario analyses that assumed population compactification and dispersion at both national and prefecture scales. The resulting population distribution projection for 2050 reveals spatial changes to population density, age structure, and the appearance of zero population areas. By overlaying these data on future LULC maps, population distribution maps can identify locations and types of MNC that will face labor shortages. Based on the results, the study proposes several political measures to overcome the challenges of the compact scenario, such as promotion of information and communications networks which enhance inter-municipal cooperation and support to invite external participants to aid in natural capital management. For the realization of the dispersed scenario, promoting industries with a high affinity for localization and developing attractive rural communities for young migrants through diversified childcare services and cultural opportunities are necessary. In conclusion, this study has developed a useful tool to support spatially explicit and practical planning for the management of natural capital at multiple scales to promote sustainable socio-ecological systems under depopulation and aging trends.

## Electronic supplementary material

Below is the link to the electronic supplementary material.

Supplementary material 1 (DOCX 5527 kb)

Supplementary material 2 (DOCX 1004 kb)

## Footnotes

1 “Region” here specifically relates to regions within Japan.

2 Fukushima prefecture was dealt with as one municipality due to the lack of municipal data resulting from the impact of the Great East Japan Earthquake and the subsequent nuclear power plant accident. IPSS also only projected prefectural future population trends for Fukushima (IPSS 2018).
