# Supplementary material for: Projecting population distribution under depopulation conditions in Japan: scenario analysis for future socio-ecological systems
Source: Sustain Sci. 2020 Aug 6;16(1):295–311. doi: 10.1007/s11625-020-00835-5 (PMC7406701; doi:10.1007/s11625-020-00835-5)
Supplement: Supplementary file 1 — Supplementary material 1 (DOCX 5527 kb) [file 11625_2020_835_MOESM1_ESM.docx]

Appendix 1-1: Results of population distribution in the nation-wide projection (middle cases)

Appendix 1-2: Results of population distribution focusing on Ishikawa prefecture (middle cases)
