# Supplementary material for: Projecting population distribution under depopulation conditions in Japan: scenario analysis for future socio-ecological systems
Source: Sustain Sci. 2020 Aug 6;16(1):295–311. doi: 10.1007/s11625-020-00835-5 (PMC7406701; doi:10.1007/s11625-020-00835-5)
Supplement: Supplementary file 2 — Supplementary material 2 (DOCX 1004 kb) [file 11625_2020_835_MOESM2_ESM.docx]

Appendix 2: Results of overlaying analysis focusing on Ishikawa prefecture

(produced capital-based compact and dispersed scenario)
